# HDL-associated ApoM is anti-apoptotic by delivering sphingosine 1-phosphate to S1P1 & S1P3 receptors on vascular endothelium

**DOI:** 10.1186/s12944-017-0429-2

**Published:** 2017-02-08

**Authors:** Mario Ruiz, Hiromi Okada, Björn Dahlbäck

**Affiliations:** 1 Department of Translational Medicine, Skåne University Hospital, Lund University, Malmö, Sweden; 2 Department of Translational Medicine, Clinical Chemistry, Wallenberg Laboratory, Lund University, Inga Marie Nilssons gata 53, SE-20502 Malmö, Sweden

**Keywords:** ApoM, Apoptosis, Endothelial cells, HDL, Lipocalins, Sphingosine 1-phospate

## Abstract

**Background:**

High-density Lipoprotein (HDL) attenuates endothelial cell apoptosis induced by different cell-death stimuli such as oxidation or growth factor deprivation. HDL is the main plasma carrier of the bioactive lipid sphingosine 1-phosphate (S1P), which it is a signaling molecule that promotes cell survival in response to several apoptotic stimuli. In HDL, S1P is bound to Apolipoprotein M (ApoM), a Lipocalin that is only present in around 5% of the HDL particles. The goal of this study is to characterize ApoM-bound S1P role in endothelial apoptosis protection and the signaling pathways involved.

**Methods:**

Human umbilical vein endothelial cells (HUVEC) cultures were switched to serum/grow factor deprivation medium to induce apoptosis and the effect caused by the addition of ApoM and S1P analyzed.

**Results:**

The addition of HDL^+ApoM^ or recombinant ApoM-bound S1P promoted cell viability and blocked apoptosis, whereas HDL^-ApoM^ had no protective effect. Remarkably, S1P exerted a more potent anti-apoptotic effect when carried by ApoM as compared to albumin, or when added as free molecule. Mechanistically, cooperation between S1P1 and S1P3 was required for the HDL/ApoM/S1P-mediated anti-apoptotic ability. Furthermore, AKT and ERK phosphorylation was also necessary to achieve the anti-apoptotic effect of the HDL/ApoM/S1P complex.

**Conclusions:**

Altogether, our results indicate that ApoM and S1P are key elements of the anti-apoptotic activity of HDL and promote optimal endothelial function.

**Electronic supplementary material:**

The online version of this article (doi:10.1186/s12944-017-0429-2) contains supplementary material, which is available to authorized users.

## Highlights

- ApoM-bound S1P and ApoM-containing HDL are anti-apoptotic.
- HDL/ApoM/S1P complex signals through S1P1 and S1P3.
- ApoM-bound S1P anti-apoptotic effect is more potent than albumin-bound S1P.

## Background

Apolipoprotein M (ApoM) is a member of the Lipocalin family and its structure is defined by an eight-stranded antiparallel β-barrel enclosing a hydrophobic binding pocket, where different ligands bind, e.g. retinol [1], oxidized phospholipids [2] and sphingosine 1-phosphate (S1P) [3]. Out of these, S1P is the only ApoM-ligand known to bind in vivo. An unusual property of ApoM is that its signal peptide is not cleaved off during secretion and used by the mature ApoM protein to anchor the protein to the phospholipid bilayer of high-density lipoproteins (HDL) [4, 5]. The plasma concentration of ApoM is approximately 0.9 μM and around 5% of all HDL particles in circulation carry ApoM and S1P [6, 7]. ApoM is the major carrier of S1P in circulation (~65%), the remaining S1P in plasma being bound to albumin (~35%) [7].

Sphingolipids have multiple key physiological functions that are important for the regulation of cell growth and survival. Ceramide and sphingosine are inducers of growth arrest and apoptosis and many stress stimuli increase the cellular levels of these compounds. In contrast, S1P is associated with suppression of apoptosis [8, 9].Five different membrane-bound, G-protein coupled S1P receptors (S1PR, S1P1-5) are known and binding of S1P to these receptors activates multiple receptor-specific downstream signaling pathways. In this way, S1P is able to regulate several biologic processes, such as immune cell trafficking, angiogenesis, cell migration and cell survival [10]. Indeed, S1PR represent important drug therapeutic targets. For instance, FTY720, also known as Fingolimod, is phosphorylated by endogenous kinases and works as a functional antagonist of S1P1 that has been approved for the treatment of multiple sclerosis [11].

The integrity of endothelial cells lining the vessels is crucial for vascular homeostasis and endothelial cell-death triggers vascular leakage and promotes inflammation in adjacent tissues [12]. Additionally, apoptotic endothelial cells become pro-coagulant and may provoke formation of blood clots [13]. Thus, increased endothelial cell apoptosis is associated with several cardiovascular pathologies, in particular with thrombosis and atherosclerosis [14].

HDL particles are potently anti-atherogenic and reduce endothelial cell apoptosis [15, 16]. Cholesterol efflux is one of the mechanisms underlying HDL protection of endothelium, and importantly, ApoM-containing HDL enhances cholesterol efflux [17, 18]. Likewise, it is known that free S1P attenuates apoptosis in endothelial cells [15, 19]. The goal of the present study was to further characterize the role of S1P in the regulation of human endothelial cell apoptosis and to define the signaling pathways involved. For that purpose, we took into account that HDL-associated S1P is bound to ApoM in plasma. We have used human ApoM-containing HDL (HDL^+ApoM^) and ApoM-lacking HDL (HDL^-ApoM^) to study regulation of apoptosis in human endothelial cells. Moreover, we have elucidated whether the anti-apoptotic properties of S1P are carrier dependent by comparing the anti-apoptotic effects of albumin-bound S1P, ApoM-bound S1P and S1P as a free molecule.

## Methods

### Cell culture and apoptosis induction

Human Umbilical Vein Endothelial Cells (HUVEC) were obtained from Gibco, grown in 1% gelatin pre-coated plates in M200 medium containing 1% penicillin and streptomycin and low serum growth supplement (LSGS) (all from Gibco) at 37 °C in a humidified 5% CO_2_ incubator. The culture medium was replaced every 2 days, and cells were subcultured at 90–95% confluence. Cells were studied between passages 2–8.

LSGS contains fetal bovine serum (FBS), human epidermal growth factor (EGF), basic fibroblast growth factor (bFGF), heparin and hydrocortisone. Removal of all these components was used to induce apoptosis in HUVEC. This treatment will be referred as serum/GF deprivation. For that, cells were washed twice with M200 medium without LSGS. The absence of S1P in M200 medium without LSGS was verified by mass spectrometry as it was previously described in [7, 20].

### Purifications (ApoM and HDL)

Recombinant soluble human ApoM (residues 22–188, without the signal peptide, Swiss-Prot entry O95445) was expressed in *E. coli*, purified from inclusion bodies and refolded as described in Ahnström et al. [1]. ApoM binding to S1P was confirmed by intrinsic fluorescence quenching and isoelectric focusing as described in Sevvana et al. [3]. ApoM loading with S1P was performed as in Ruiz et al.[21].

HDL was isolated from human plasma obtained from the Blood Bank at Växjö Hospital, Sweden, as described in Ruiz et al. [21]. Briefly, HDL were separated by ultracentrifugation followed by size exclusion chromatography. HDL^+ApoM^ and HDL^-ApoM^ were isolated by immunoaffinity chromatography with M23 and M58 monoclonal antibodies against ApoM.

S1P levels in HDL preparations were quantified by mass spectrometry as it was previously described [7, 20]. S1P was ~0.146 μM/mg protein in total HDL, ~0.417 μM/mg of protein in HDL^+ApoM^ and ~0.008 μM/mg protein in HDL^-ApoM^.

### Protein quantification, protein electrophoresis and western blot

Sample protein concentration was quantified using BCA protein assay kit (Pierce) according manufacturer’s instructions.

Electrophoresis was done in 4–15% gradient pre-casted SDS-gels (Bio-Rad) under reducing conditions. Western blotting was done after separation in a Trans-Blot Turbo transfer system (Bio-Rad). An Antibody against phospho-ERK1 (T202/Y204) / phospho-ERK2 (T185/Y187) ERK1/2 was from R&D systems; antibodies against total ERK (#9102), pSer473 AKT (D9E), total AKT (C67E7) were from Cell Signaling and an antibody against GAPDH was from Santa Cruz Biotechnology (#20357).

### Annexin V staining and flow cytometry

Cells were detached with TrypLE Express (Gibco), washed and resuspended in Annexin V binding buffer (BD Bioscience). Then, cells were stained with PE Annexin V and 7-ADD according manufacturer’s instructions (BD Bioscience) and analyzed in a Cytomics FC500 (Beckman Coulter) flow cytometer. Data were analyzed with FlowJo X v.10.0 7r2. Early apoptotic cells were defined by Annexin V^+^ and 7-ADD^−^.

### Measurement of caspase-3 activity

Caspase-3 activity was measured using a colorimetric assay kit according to manufacturer’s instructions (Abcam). Briefly, cell lysates (50 μg total protein) were incubated in the presence of N-acetyl-Asp-Glu-Val-Asp-p-nitroanilide (Ac-DEVD-pNA, 200 μM) and the release of pNA was measured using a plate reader (TECAN Infinite F200) at 405 nm.

### Cell viability assay

Cell viability was evaluated by the MTT assay following manufacturer’s instructions (Roche). Briefly, viable cells are defined by their ability to reduce MTT (3-(4,5-dimethylthiazol-2-yl)-2,5-diphenyltetrazolium bromide) to formazan, which is a measure of an active metabolism. The conversion was quantified using a plate reader (TECAN Infinite F200) at 570 nm and optical density value was utilized as an indicator of cell viability.

### Quantitative real-time PCR (qPCR)

Total cellular RNA was isolated using RNeasy Kit according to the manufacturer’s instructions (Qiagen) and quantified using a NanoDrop spectrophotometer (ND2000, Thermo Scientific). qPCR were performed with a CFX384 C1000 thermal cycler (Bio-Rad) using the Super Scrip III Platinum One Step qRT-PCR kit (Invitrogen) and TaqMan probes (Applied Biosystems): 4326317E (*GAPDH*), Hs00173499_m1 (S1P1), AJ39RQ5 (S1P2), Hs00245464_s1 (S1P3), Hs02330084_s1 (S1P4) and Hs00928195_s1 (S1P5) according manufacturer’s instructions. Samples were measured as quadruplicates. The relative expression of each gene was calculated according to the ΔΔCT method [22]. Expression of the housekeeping gene GAPDH was used to normalize for variations in RNA input.

### Other reagents

Sphingosine-1-Phosphate (d18:1; Lipid Maps LMSP01050001) was purchased from Avanti Polar Lipids and Sigma; bovine fatty acid free albumin was from Sigma; W146, CAY10444 and ML-031 were from Cayman Chemical; SEW2871 and CYM5541 were from Tocris Bioscience; LY294002, U0126 and PD98059 were from R&D systems.

### Statistical analysis

Statistical analyses were performed with SigmaPlot 11.0 software (Systat Software Inc.). A value of *p* < 0.05 was defined as threshold for significant changes. Student *t*-test and Mann-Whitney *U*-test were used for two-sample comparisons and ANOVA was used when assaying for multiple comparisons. The particular tests used for *post hoc* analyses depended on homoscedasticity, and are stated in the figure legends.

## Results

### HDL^+ApoM^ protects endothelial cells against apoptosis and promotes cell survival

**Fig. 1:**
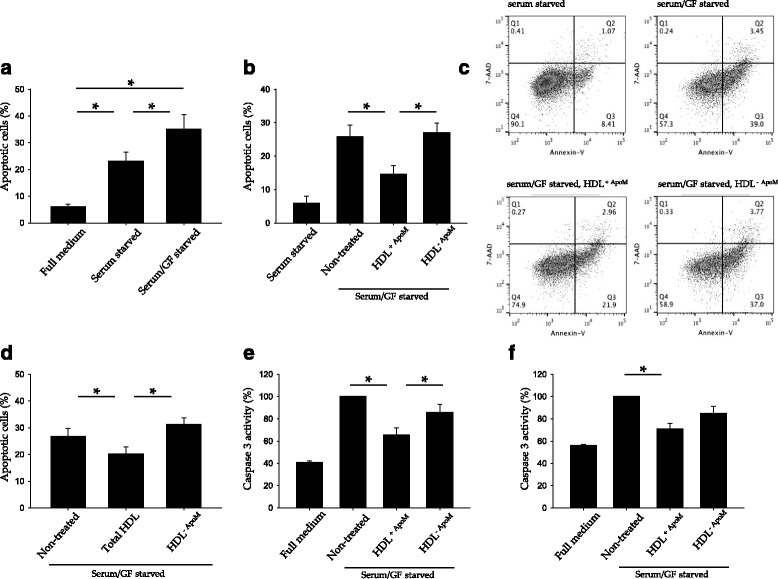
HDL containing ApoM protects endothelial cells against serum/GF deprivation-induced cell death. **a** HUVEC were grown to confluence in full medium and then switched to serum starvation medium or serum/GF deprivation medium. The graph represents the percentage of apoptotic cells (Annexin V^+^ and 7-ADD^−^) identified by flow cytometry. *Error* bars correspond to SEM of *n* = 5. One-way ANOVA *p* < 0.001 followed by Holm-Sidak method multiple-comparison *post hoc* test. **b**–**d** Cells were serum/GF deprived and treated with ± HDL^+ApoM^ 50 μg/ml or HDL^-ApoM 50^ μg/ml in **b** and **c** and ± Total HDL 500 μg/ml or HDL^-ApoM^ 500 μg/ml in **d** for 18 h and then analyzed by flow cytometry. Error bars correspond to SEM. **c** shows dot plots from a representative experiment of B. In **b**, *n* = 4, one-way ANOVA *p* = 0.001 followed by Holm-Sidak method multiple-comparison *post hoc* test. In **d**, *n* = 10, ANOVA on ranks *p* = 0.016 followed by SNK method multiple-comparison *post hoc* test. **e**-**f** Measurements of Caspase-3 activity in HUVEC lysates. Cells were incubated in serum/GF starvation medium with ± HDL^+ApoM^ 25 μg/ml, HDL^-ApoM^ 25 μg/ml for 24 h in **e** and for 2 h in **f**. Data were normalized versus serum/GF starvation condition and error bars correspond to SEM. In E, *n* = 4, ANOVA on ranks *p* = 0.006 followed by SNK method multiple-comparison *post hoc* test. In F, *n* = 3, one-way ANOVA *p* = 0.012 followed by Holm-Sidak method multiple-comparison *post hoc* test. *p < 0.05

Endothelial cells undergo apoptosis when deprived of serum and growth factors (Fig. 1a) [15, 16, 23]. However, HDL addition to the cell medium mitigates serum/GF deprivation induced cell death [15, 16]. To assess the role of ApoM and S1P in HDL mediated protection we isolated HDL^+ApoM^ and HDL^-ApoM^. Then, HUVEC were serum/GF deprived in the presence of HDL^+ApoM^ or HDL^-ApoM^ for 18 h and the amount of apoptotic cells measured by flow cytometry. HDL^+ApoM^ reduced the percentage of apoptotic cells, whereas HDL^-ApoM^ did not confer any protection against serum/GF deprivation (Fig. 1b and c). Consistently, total HDL also protected HUVEC against serum/GF deprivation (Fig. 1d). To confirm the anti-apoptotic effect of HDL^+ApoM^, we measured Caspase-3 activity in HUVEC after 24 h of serum/GF deprivation. Caspase-3 activity in cultures treated with HDL^+ApoM^ upon serum/GF deprivation was significantly lower than in cultures treated with HDL^-ApoM^ or without HDL (Fig. 1e). Next, we investigated whether the anti-apoptotic effect of HDL^+ApoM^ could also be achieved after a short serum/GF deprivation time. Therefore, we quantified Caspase-3 activity 2 h after the removal of serum and growth factors and found a reduction of Caspase-3 activity in lysates from HDL^+ApoM^ treated cells, whereas HDL^-ApoM^ treatment did not confer protection against serum/GF deprivation induced cell-death (Fig. 1f).

**Fig. 2:**
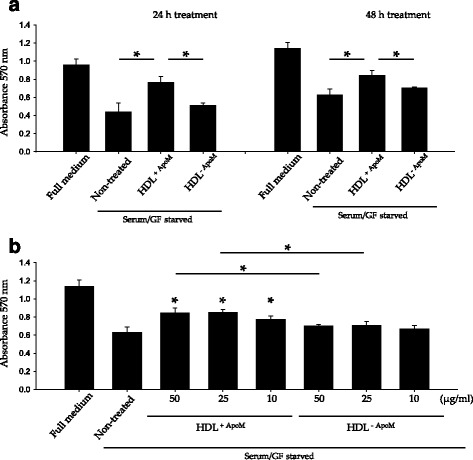
HDL containing ApoM promotes endothelial cell viability upon serum/GF deprivation. **a** MTT assay of HUVEC after 24 h (*left*) or 48 h (*right*) of incubation in serum/GF deprivation medium with or without HDL^+ApoM^ 50 μg/ml or HDL^-ApoM^ 50 μg/ml. Data are expressed as mean ± SD. *N* = 4, one-way ANOVA *p* < 0.001 followed by Holm-Sidak method multiple-comparison *post hoc* test. **b** Cells were assayed as in **a**, but HDL^+ApoM^ or HDL^-ApoM^ concentrations were 50, 25 or 10 μg/ml. Data are expressed as mean ± SD. *N* = 4, one-way ANOVA *p* < 0.001 followed by Holm-Sidak method multiple-comparison *post hoc* test. * *over* the bars indicates statistical significance versus serum/GF deprivation treatment. *p < 0.05

Since the HDL^+ApoM^ treatment of HUVECs is anti-apoptotic, it is expected to have higher cell viability in those cultures. We verified this hypothesis by using the MTT assay. Serum/GF deprivation reduced HUVEC viability, but this reduction was significantly mitigated by HDL^+ApoM^. In contrast, HDL^-ApoM^ did not improve cell viability either after 24 h or after 48 h of serum/GF deprivation (Fig. 2a). Next, we investigated which concentration of HDL^+ApoM^ was required to promote cell viability upon serum/GF deprivation. Interestingly, HDL^+ApoM^ at 50 μg/ml and 25 μg/ml significantly increased cell viability when compared to HDL^-ApoM^ and non-HDL treatments, whereas HDL^+ApoM^ at 10 μg/ml only significantly increased cell-viability when compared to non-HDL treatment (Fig. 2b).

Thus, we conclude that the HDL anti-apoptotic effect in serum/GF deprived endothelial cells is primary mediated by HDL containing ApoM and S1P.

### S1P1 and S1P3 activation mediate the protective effect of ApoM-associated HDL

**Fig. 3:**
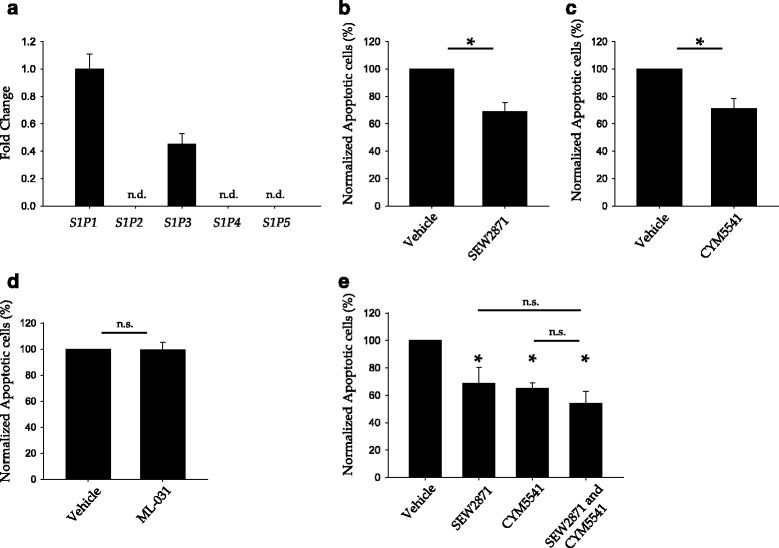
Pharmacological activation of S1P1 or S1P3 protects endothelial cells against serum/GF deprivation-induced cell death. **a** Relative expression of S1PR in HUVEC. Total RNA was analyzed by qPCR using Taqman probes for S1PR and normalized against *GAPDH* expression. *S1P1* expression was chosen as reference. *Error* bars correspond to SD, n.d., not detected. **b**–**e** HUVEC were grown up to confluence in full medium, then switched to serum/GF deprivation medium with SEW2971 5 μM **b** and **e**, ML-031 5 μM **c** and **e** or/and CYM5541 5 μM **d** and **e** for 18 h and then analyzed by flow cytometry. The percentage of apoptotic cells (AnnexinV^+^ and 7-ADD^−^) was normalized versus serum/GF starvation condition. *Error* bars correspond to SEM. In **b**, *n* = 9, Mann-Whitney *U*-test *p* <0.001. In **c**, *n* = 7 Mann-Whitney *U*-test *p* = 0.026. In D, *n* = 4, Mann-Whitney *U*-test *p* = 0.343. In **e**, *n* = 5, one-way ANOVA *p* = 0.004 followed by Holm-Sidak multiple-comparison *post hoc* test. In **e**, * *over* the bars indicates statistical significance versus serum/GF deprivation treatment. *p < 0.05

S1P signals through five different G-couple protein receptors known as S1P1-5. Thus, to understand the anti-apoptotic role of HDL^+ApoM^ in the endothelium, we studied the expression of S1PR in HUVEC by qPCR. We found that HUVEC express *S1P1* and *S1P3*, but do not express *S1P2*, *S1P4* or *S1P5* (Fig. 3a). Since *S1P2* expression in HUVEC has been reported previously [24], we simultaneously run a qPCR using HEK293 cDNA as a positive control of S1P2 expression to assure the correct performance of S1P2 probe (data not shown). Then, we examined which S1P receptor/s are responsible for HDL^+ApoM^ anti-apoptotic function. For that purpose, we followed a pharmacological approach and used receptor-specific agonists to mimic S1P stimulation. SEW2871, an S1P1 specific agonist, and CYM5541, an S1P3 specific agonist, reduced the amount of apoptotic endothelial cells upon serum/GF deprivation (Fig. 3b and c respectively). We also tested the S1P2 specific agonist ML-031. Nevertheless, ML-031 did not confer any protection against apoptosis (Fig. 3d). Next, we investigated if simultaneous pharmacological activation of S1P1 and S1P3 could confer a greater protection against serum/GF deprivation. However, the percentage of apoptotic cells treated with both, SEW2871 and CYM5541, is comparable to the cells only treated with SEW2871 or CYM5541 (Fig. 3e).

**Fig. 4:**
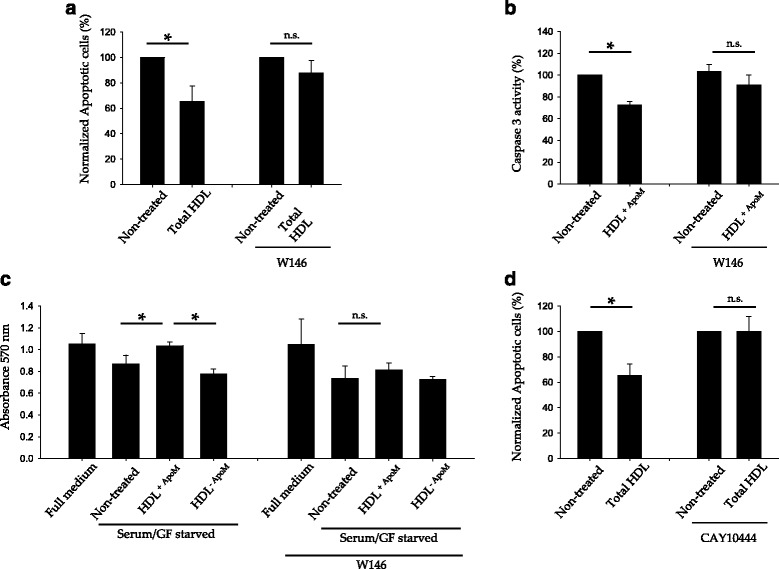
S1P1 and S1P3 activation mediates the protective effect of ApoM-associated HDL in human endothelium. **a**-**d** Cells were pre-incubated with ± W146 1 μM or CAY10444 5 μM in serum/GF starvation medium for 30 min. **a** HUVEC were incubated in serum/GF starvation medium with total HDL 500 μg/ml and W146 1 μM for 18 h and AnnexinV^+^ and 7-ADD^−^ cells were quantified by flow cytometry. As control, the experiment was replicated without W146. *Error* bars indicated SEM of *n* = 4, Mann-Whitney *U*-test *p* = 0.029 when W146 was absent and Mann-Whitney *U*-test *p* = 0.343 in W146 presence. **b** Cells were treated with HDL^+ApoM^ 25 μg/ml plus W146 1 μM for for 2 h and then lysated and Caspase-3 activity measured. Data are represented as the mean ± SEM of *n* = 2–6. Student *t*-test *p* = 0.010 when W146 was not added; Student *t*-test *p* = 0.388 in W146 presence. **c** Cell viability was assayed by MTT. Cells were switched to serum/GF starvation medium with ± HDL^+ApoM^ 25 μg/ml, HDL^-ApoM 25^ μg/ml and ± W146 1 μM for 24 h. *Error* bars represent SD of *n* = 3. Control condition (no W146) data were analyzed by one-way ANOVA *p* = 0.004 followed by Holm-Sidak method multiple-comparison *post hoc* test. W146 condition data were analyzed by ANOVA on ranks *p* = 0.059. **d** Cells were assayed as in A, but using CAY10444 5 μM instead of W146. *Error* bars indicated SEM of *n* = 4-5, Mann-Whitney *U*-test, *p* = 0.029 when CAY10444 was not present: Mann-Whitney *U*-test *p* = 0.690 in CAY10444 presence. *p < 0.05

To confirm the participation of S1P1 and S1P3 in HDL containing ApoM protection against serum/GF deprivation, we used S1P1 and S1P3 specific antagonists. Blockage of S1P1 with W146 abolished the anti-apoptotic effect of total HDL or HDL^+ApoM^ in serum/GF deprived HUVEC (Fig. 4a and b, respectively). Additionally, W146 also abrogated the increment of viability caused by HDL^+ApoM^ in serum/GF deprived HUVEC (Fig. 4c). Likewise, blockage of S1P3 with CAY10444 abolished the anti-apoptotic effect of total HDL in serum/GF deprived HUVEC (Fig. 4d).

In conclusion, HDL required S1P1 and S1P3 signaling to achieve their anti-apoptotic effect in serum/GF deprived HUVEC. However, pharmacological activation of S1P1 or S1P3 was sufficient to mimic HDL protection.

### ApoM-bound S1P confers longer protection to endothelial cells against serum/GF deprivation

**Fig. 5:**
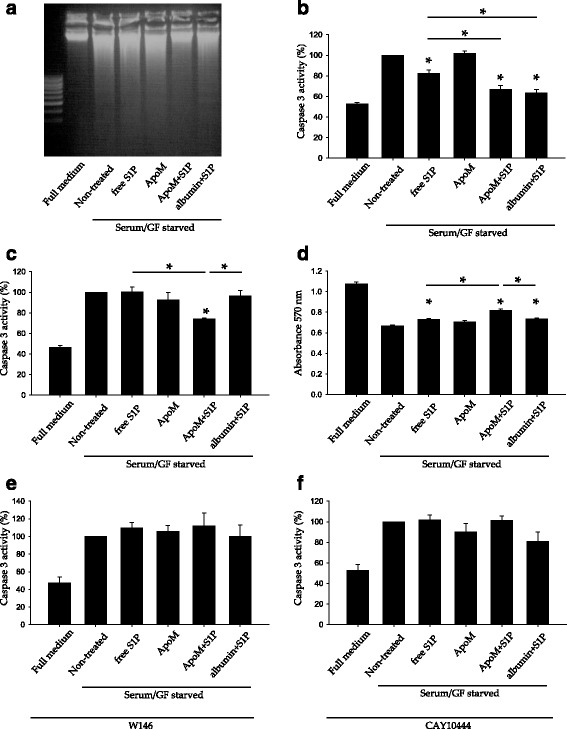
ApoM-bound S1P confers longer protection to endothelial cells against serum/GF deprivation. **a**–**e** Cells were treated with free S1P 1 μM, ApoM 1 μM, ApoM-bound S1P 1 μM or albumin-bound S1P 1 μM. **a** Cells were treated for 24 h and then a cell fragmentation assay performed. **b** Cells were treated for 2 h and then the Caspase 3 activity measured. ANOVA on ranks *p* < 0.001 followed by SNK multiple-comparison *post hoc* test. **c** As **b**, but 24 h treatment. ANOVA on ranks *p* = 0.007 followed by SNK multiple-comparison *post hoc* test. **d** Cell viability was determined by MTT after 24 h of treatment. *Error* bars correspond to SD. *N* = 4-5. One-way ANOVA *p* < 0.001 followed by Holm-Sidak method multiple-comparison *post hoc* test. **e**-**f** Cells were pre-incubated with ± W146 1 μM or CAY10444 10 μM in serum/GF deprivation medium for 30 min. and then treated as in **c**, but with the addition of W146 1 μM in **e** and CAY10444 10 μM in F. In E, *n* = 3, data were analyzed by one-way ANOVA *p* = 0.003 followed by Holm-Sidak method multiple-comparison *post hoc* test and in F, *n* = 3, by One-way ANOVA *p* < 0.001 followed by Holm-Sidak method multiple-comparison *post hoc* test. * *over* the bars indicates statistical significance versus serum/GF starvation treatment. *p < 0.05

Plasma S1P is mostly carried by ApoM in HDL, but a fraction is bound to albumin [7]. Therefore, we elucidated if albumin-bound S1P could also protect endothelial cells against serum/GF deprivation induced cell-death. In order to have a direct comparison between ApoM-bound S1P and albumin-bound S1P, we produced soluble recombinant human ApoM in *E. coli* and loaded it with S1P. Previous work has studied S1P in apoptosis by directly adding S1P as a free molecule to the cell medium (for instance [9, 25–27]). Therefore, we also included free S1P in our study. First, as a visual approximation, we performed a DNA fragmentation assay. Endothelial cells were serum/GF deprived for 24 h in the presence of free S1P, ApoM, ApoM-bound S1P or albumin-S1P. Interestingly, ApoM-bound S1P treated cells showed a lower level of DNA fragmentation than free S1P or albumin-bound S1P treated cells (Fig. 5a). To confirm this result we carried out Caspase-3 activity assays. Importantly, free S1P, ApoM-bound S1P and albumin-bound S1P decreased Caspase-3 activity after 24 h of serum/GF deprivation (Fig. 5b). However, ApoM-bound S1P and albumin-bound S1P did it more efficiently than free S1P. Remarkably, when we looked at more prolonged protection, 48 h of serum/GF deprivation, only ApoM-bound S1P reduced Caspase-3 activity in HUVEC upon serum/GF deprivation (Fig. 5c). In consonance with this finding, free S1P, ApoM-bound S1P and albumin-bound S1P treatments improved cell viability upon serum/GF deprivation, but ApoM-bound S1P was significantly more effective than free S1P and albumin-bound S1P (Fig. 5d).

Since anti-apoptotic and pro-survival effects of S1P were carrier dependent, we investigated if differences can be due to particular activation of S1PR. To study this, we performed Caspase-3 assays as in Fig. 5b, but in the presence of the S1P1 antagonist W146 or the S1P3 antagonist CAY10444. Interestingly, all three alternative ways to supply S1P to endothelial cells required S1P1 and S1P3 signaling to become anti-apoptotic (Fig. 5e and f).

Thus, we concluded that the anti-apoptotic effect of S1P in serum/GF deprived endothelial cells was carrier dependent, ApoM-bound S1P being the most powerful of all three carriers. Furthermore, anti-apoptotic activity of S1P was mediated by S1P1 and S1P3 with independence of which S1P carrier was used.

### PI3K/AKT and ERK1/2 signaling pathways are implicated in the anti-apoptotic effect of S1P in serum/GF deprived cells

**Fig. 6:**
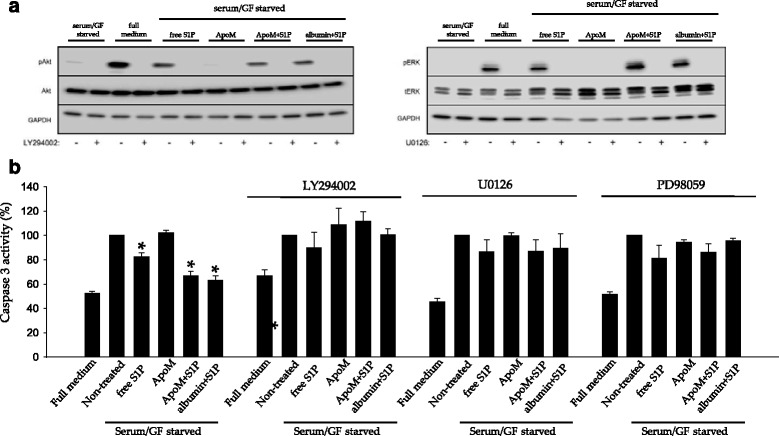
AKT or ERK inhibition blocks S1P anti-apoptotic effect. **a**-**b** Cells were pre-incubated with ± W146 1 μM, CAY10444 10 μM, LY294002 50 μM, U0126 50 μM or PD98050 50 μM for 30 min and then treated with free S1P 1 μM, ApoM 1 μM, ApoM-bound S1P 1 μM or albumin-bound S1P 1 μM for 10 min in **a** or 2 h in **b**. In **a**, western *blots* to analyze the phosphorylation of AKT and ERK induced by the treatments mentioned *above*. Total AKT, ERK and GAPDH were analyzed as controls. In B, Caspase-3 activity in cell lysates after the different treatments indicated *above*. Data are expressed as mean ± SEM of *n =* 3–6. No inhibitor set: ANOVA on ranks *p* < 0.001 followed by SNK method multiple-comparison *post hoc* test. LY294002 set: one-way ANOVA *p* = 0.010 followed by Holm-Sidak method multiple-comparison *post hoc* test. U0126 set: ANOVA on ranks *p* = 0.018 followed by Dunn’s method multiple-comparison *post hoc* test. PD98050 set: one-way ANOVA *p* < 0.001 followed by Holm-Sidak method multiple-comparison *post hoc* test. *p < 0.05

It has been shown that the anti-apoptotic activities of S1P and HDL are mediated by PI3K/AKT and ERK1/2 signaling pathways [15, 16, 28]. Moreover, a previous study demonstrated that phosphorylation of AKT and ERK is induced by HDL^+ApoM^ and albumin-S1P, but not by HDL^-ApoM^ [7]. To link these antecedents, we used the PI3K/AKT inhibitor LY249002 and the MEK inhibitors U0126 and PD98059. We added these inhibitors to serum/GF deprived cells in the presence of free S1P, ApoM, ApoM-bound S1P or albumin-bound S1P. Then, we lysed the cells and analyzed them by western-blot or Caspase-3 activity., The phosphorylation of AKT and ERK by free S1P, ApoM-bound S1P or albumin-S1P was abolished when cells were treated with LY249002 or U0126 (Fig. 6a). LY294002, U0126 and PD98059 also canceled the inhibitory effect of free S1P, ApoM-bound S1P or albumin-S1P on Caspase-3 activation in serum/GF deprived HUVEC (Fig. 6b).

**Fig. 7:**
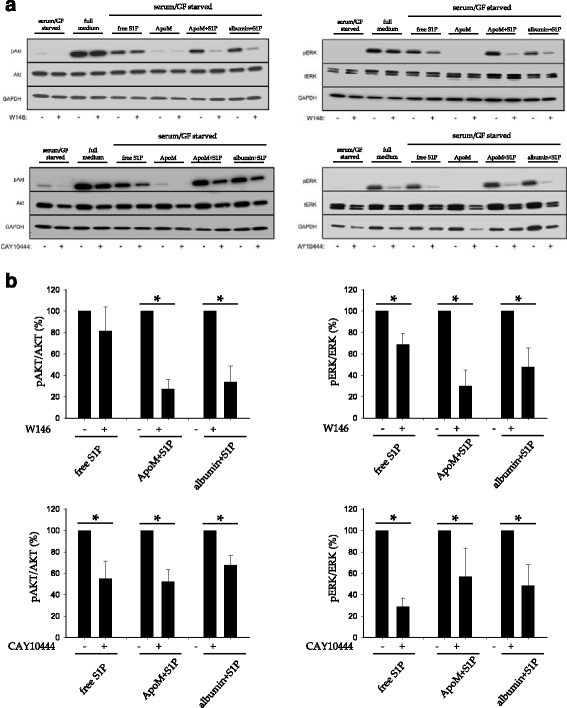
S1P1 or S1P3 antagonists block AKT and ERK phosphorylation. **a** Cells were pre-incubated with ± W146 1 μM or CAY10444 10 μM for 30 min and then treated with free S1P 1 μM, ApoM 1 μM, ApoM-bound S1P 1 μM or albumin-bound S1P 1 μM for 10 min. Then, cells were lysed and analyzed by western blot for pAKT and pERK. Total AKT, ERK and GAPDH were analyzed as controls. **b** Quantification of relevant pairs of A. Student *t*-test of *n* = 3 independent experiments. Error bars correspond to SD. *p < 0.05

Next, we determined whether S1PR activation mediated the phosphorylation of AKT and ERK by S1P. The selective S1P1 antagonist W146 dramatically reduced the phosphorylation of AKT and ERK by ApoM-bound S1P and albumin-bound S1P. However, when S1P was added as a free molecule, W146 decreased ERK phosphorylation but surprisingly not AKT phosphorylation. In contrast, CAY10444 reduced the phosphorylation of AKT and ERK mediated by free S1P, ApoM-bound S1P and albumin-S1P (Fig. 7a and b).

In conclusion, S1P anti-apoptotic effect on serum/GF deprived endothelial cells went via S1P1 and S1P3 and required the phosphorylation of AKT and ERK1/2 (Additional file 1: Fig. S1).

## Discussion

Previous studies have pointed out the protective role of HDL on endothelial cells upon different cell-death stimuli, including oxidized LDL [29, 30] and serum/GF deprivation [15, 16]. Likewise, anti-apoptotic properties of free S1P have been demonstrated [9, 25–27, 30, 31]. Here we connect previous findings and show that ApoM-containing HDL, and therefore S1P, have anti-apoptotic and pro-survival properties in serum/GF deprived endothelial cells (Figs. 1 and 2). Importantly, S1P also promotes survival in cardiomyocytes [32], macrophages [31] and other cell types [33–36]. Now, it would be relevant to study S1P protection in other human cell types taking in account ApoM. It is important to highlight that HDL particles are highly heterogenic in protein and lipid composition and additional cytoprotective mechanisms are possible [37]. Which ones are relevant may depend on the cell-death stimulus, time and concentration used.

de Souza et al. [29] isolated HDL subpopulations and found that small and dense HDL3, which are enriched in S1P [and ApoM, [38]], have cytoprotective activity superior to that of large and light HDL2. Interestingly, reconstituted HDL (rHDL) with added S1P did not enhance the anti-apoptotic effect achieved by rHDL without S1P [29]. Similarly, S1P-fortified HDL subfractions did not to significantly improve the anti-apoptotic effect of non-S1P-fortified HDL. Both scenarios could be explained by the fact that the exogenous S1P was not bound to ApoM and therefore may not properly interact with S1PR. This explanation concurs with Fig. 5a-d, where ApoM-S1P displayed significantly elevated anti-apoptotic activity as compared to free S1P or albumin-S1P. In agreement, apoptosis was not inhibited when albumin-S1P was used at 1–100 nM [29]. Likewise, rHDL anti-apoptotic ability is enhanced when plasmalogens are incorporated to rHDL [39], but the molecular mechanism behind has not been described yet. Several endothelial cell types express ApoM [40] and the S1P transporter Spns2 [41]. Possibly, rHDL including plasmalogens are better acceptors for ApoM and S1P than plasmalogen-free rHDL.

Riwanto et al. [42] demonstrated that ApoJ enhances HDL anti-apoptotic effect on endothelial cells. However, ApoJ is absent in our HDL^+ApoM^ preparations [17] and, therefore, ApoM-S1P anti-apoptotic effect cannot be ascribed to ApoJ. In contrast, HDL anti-apoptotic activity is impaired in HDL enriched in ApoC-III [42], which it is less abundant in HDL^+ApoM^ than in HDL^-ApoM^ [17]. Thus, the poor anti-apoptotic capacity of ApoC-III containing HDL can be explained by the low content in ApoM-S1P.

Endothelial-cell survival is enhanced by free S1P via S1P1 and S1P3 [19]. We corroborated this finding and demonstrated that parallel activation of both S1P1 and S1P3 by HDL^+ApoM^ is required to achieve S1P anti-apoptotic and pro-survival effects (Figs. 4 and 5). Furthermore, we show that S1P1 and S1P3 activation requirement is independent of the S1P carrier (Fig. 5e-f). However, activation by ApoM-S1P renders a longer protection than albumin-S1P. These apparently conflicting data can be explained by S1P carrier specific degradation of S1P1 [43, 44]. Following activation of S1P1 by albumin-S1P, S1P1 is internalized and degraded by the proteasome, whereas S1P1 is internalized and recycled to the plasma membrane after ApoM-S1P activation. Unfortunately, no data on S1P-carrier dependent biology of S1P3 are available. However, an analogous situation to S1P1 may be plausible for S1P3.

Beyond S1PR, other plasma membrane receptors connect apoptosis, HDL and its major component, ApoA1. First, HDL3 acts via Scavenger Receptor Class B Type I (SR-BI) to inhibit apoptosis on endothelial cells [45]. Indeed, Li et al. [46] over-expressed SR-BI in CHO cells and elaborated an attractive model in which SR-BI is a pro-apoptotic receptor in absence of HDL. This model needs to be validated in endothelial cells, but the fact that HDL^+ApoM^ are more efficient than HDL^-ApoM^ in stimulating cholesterol efflux suggests that HDL^+ApoM^ may have higher affinity for SR-BI than in HDL^-ApoM^. Additionally, stimulation of F1-ATPase by lipid-free ApoA1 inhibits endothelial cell apoptosis [45], but interactions between HDL and F1-ATPase have not been reported.

AKT and ERK1/2 phosphorylation mediate HDL and S1P cytoprotective actions [15, 16, 27, 28]. Moreover, HDL^+ApoM^ and albumin-S1P, but not HDL^-ApoM^, phosphorylate AKT and ERK [7]. Here, we confirmed S1P-dependent phosphorylation of ERK and AKT and demonstrated that blockage of AKT and ERK signaling abolishes S1P anti-apoptotic effects (Fig. 6). Importantly, activation of S1P1 and S1P3 by ApoM-S1P or albumin-S1P phosphorylate AKT and ERK (Fig. 7). Interestingly, S1P induces AKT activation and protects against ischemia/reperfusion in mouse cardiomyocytes via S1P2 and S1P3 [47, 48]. This suggests that the pattern of S1PR activated by S1P to achieved cytoprotection may be tissue/organism dependent.

Retinol binding protein (RBP) is another member of the Lipocalin family and transports retinol in plasma. Interestingly, apo-RBP is pro-apoptotic, whereas holo-RBP is anti-apoptotic [49]. We did not identify any pro-apoptotic activity of apo-ApoM *per se* in endothelial cells. However, ApoM over-expression promotes apoptosis in the human hepatoma derived cell line HepG2 [50]. Moreover, two other plasma Lipocalins: Lipocalin-type prostaglandin D2 synthase (L-PGDS) and Apolipoprotein D (ApoD) mitigate cell-death and promote viability [51, 52]. Interestingly, ApoM and ApoD are HDL enriched in HDL3 [38]. Thus, abundance of Lipocalins in HDL3 could explain the high cytoprotective ability of HDL3.

## Conclusions

Taken together, our results demonstrate that the HDL/ApoM/S1P-complex plays an essential role in vascular biology and protects endothelial cells from apoptosis. This is especially relevant in pathologies where endothelial cell apoptosis is altered such as in thrombosis and atherosclerosis.

## Additional file

Additional file 1: Schematic representation summarizing the anti-apoptotic effect of ApoM-bound S1P in endothelial cells. (PPTX 631 kb)

## Abbreviations

ApoM: Apolipoprotein M
HDL: High-density lipoproteins
HDL^+ApoM^: ApoM-containing HDL
HDL^-ApoM^: ApoM-lacking HDL
HUVEC: Human umbilical vein endothelial cells
S1P: Sphingosine 1-phosphate
S1PR: S1P receptor
