# Supplementary material for: HDL-associated ApoM is anti-apoptotic by delivering sphingosine 1-phosphate to S1P1 & S1P3 receptors on vascular endothelium
Source: Lipids Health Dis. 2017 Feb 8;16:36. doi: 10.1186/s12944-017-0429-2 (PMC5299634; doi:10.1186/s12944-017-0429-2)
Supplement: Additional file 1: — Schematic representation summarizing the anti-apoptotic effect of ApoM-bound S1P in endothelial cells. (PPTX 631 kb) [file 12944_2017_429_MOESM1_ESM.pptx]

## Slide 1
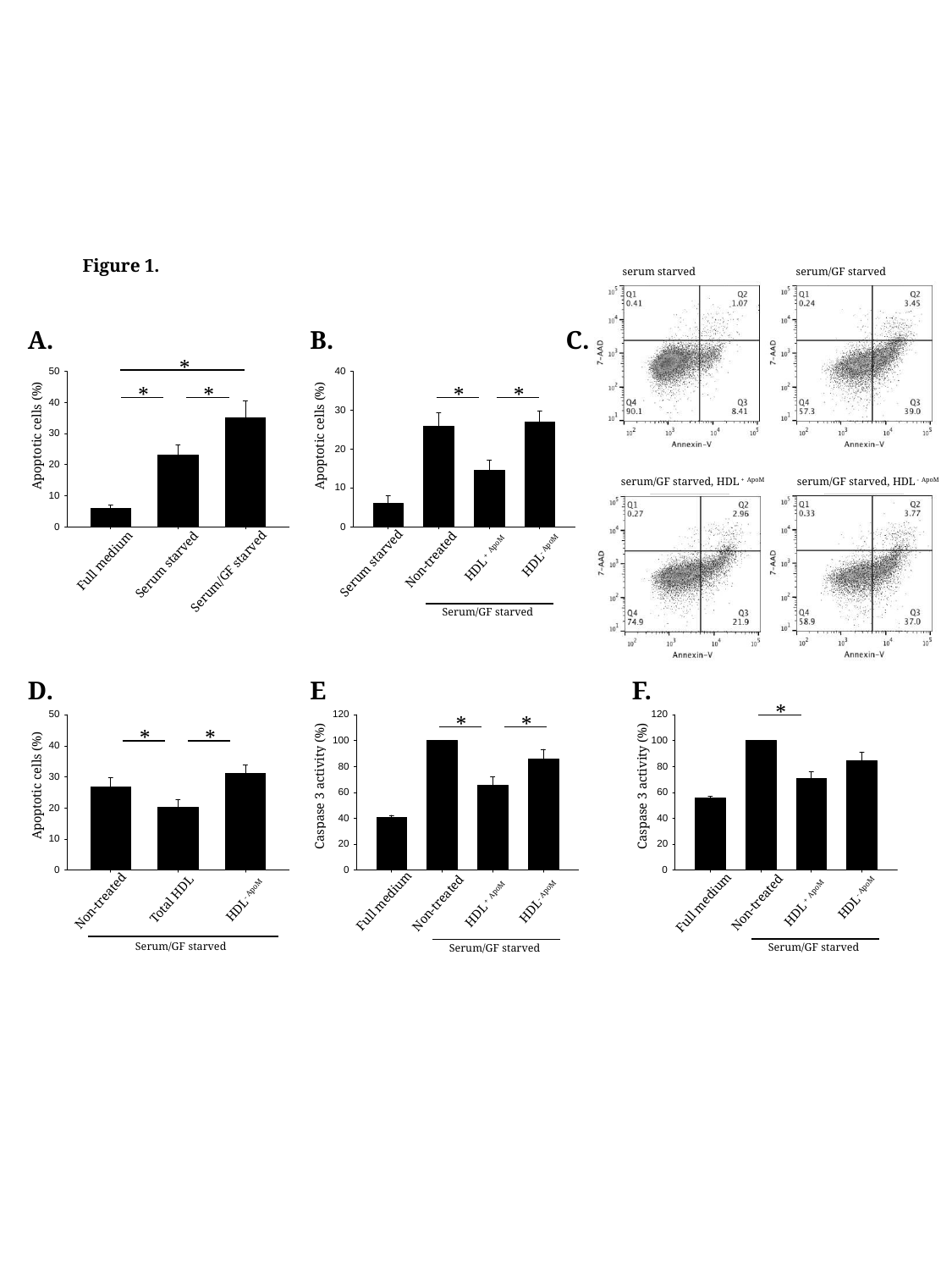

Figure 1.
serum starved
serum/GF starved
C.
serum/GF starved, HDL + ApoM
serum/GF starved, HDL - ApoM
A.
B.
*
*
*
*
*
Apoptotic cells (%)
Apoptotic cells (%)
HDL + ApoM
HDL- ApoM
Non-treated
Full medium
Serum starved
Serum starved
Serum/GF starved
Serum/GF starved
D.
E.
F.
*
*
*
*
*
*
*
Apoptotic cells (%)
Caspase 3 activity (%)
Caspase 3 activity (%)
Total HDL
HDL- ApoM
HDL- ApoM
HDL + ApoM
HDL + ApoM
HDL- ApoM
Non-treated
Non-treated
Non-treated
Full medium
Full medium
Serum/GF starved
Serum/GF starved
Serum/GF starved

## Slide 2
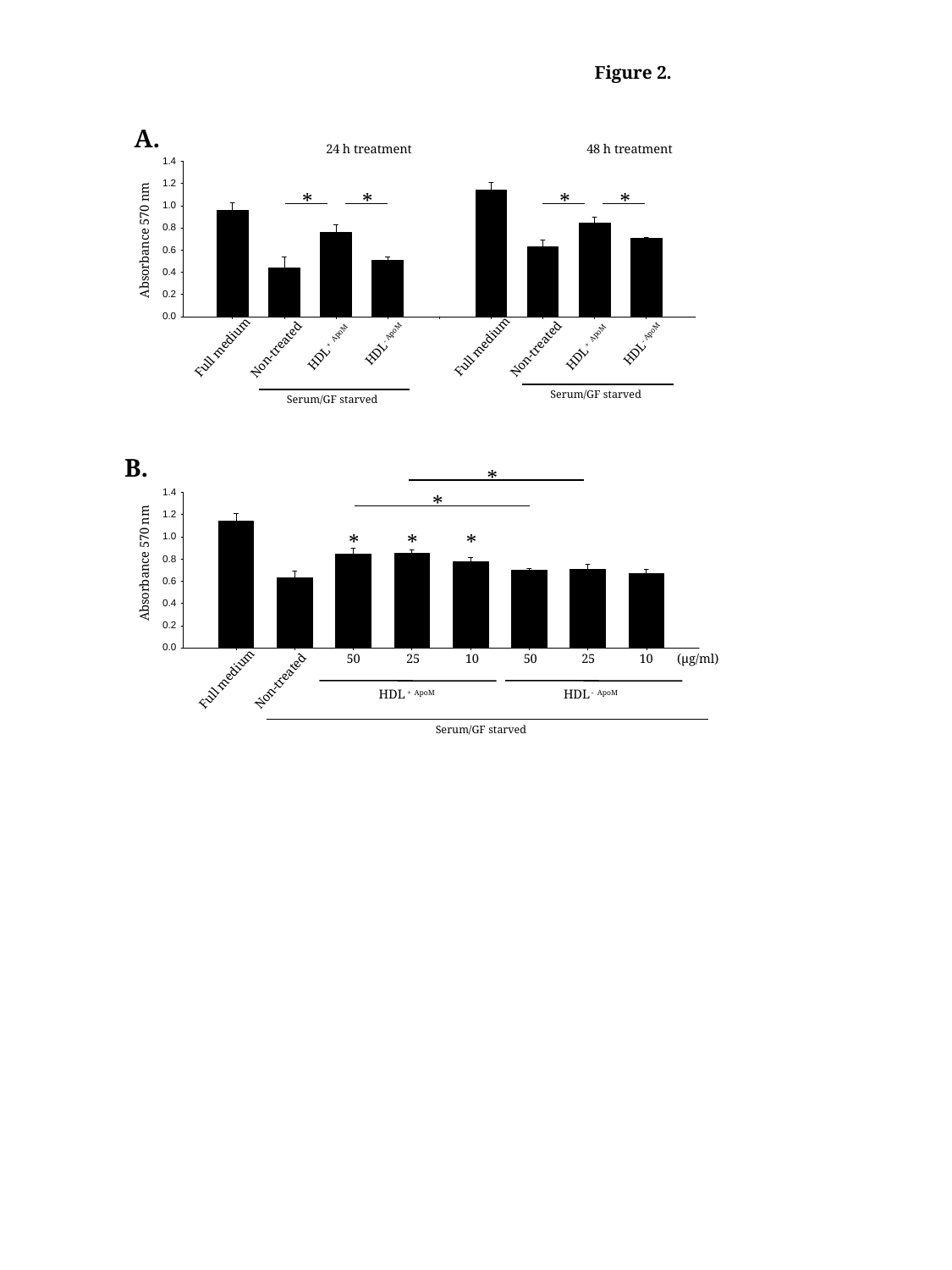

Figure 2.
A.
24 h treatment
48 h treatment
*
*
*
*
Absorbance 570 nm
HDL + ApoM
HDL- ApoM
HDL + ApoM
HDL- ApoM
B.
*
*
*
*
*
Absorbance 570 nm
50
25
10
50
25
10
(µg/ml)
HDL + ApoM
HDL - ApoM
Non-treated
Non-treated
Full medium
Full medium
Serum/GF starved
Serum/GF starved
Non-treated
Full medium
Serum/GF starved

## Slide 3
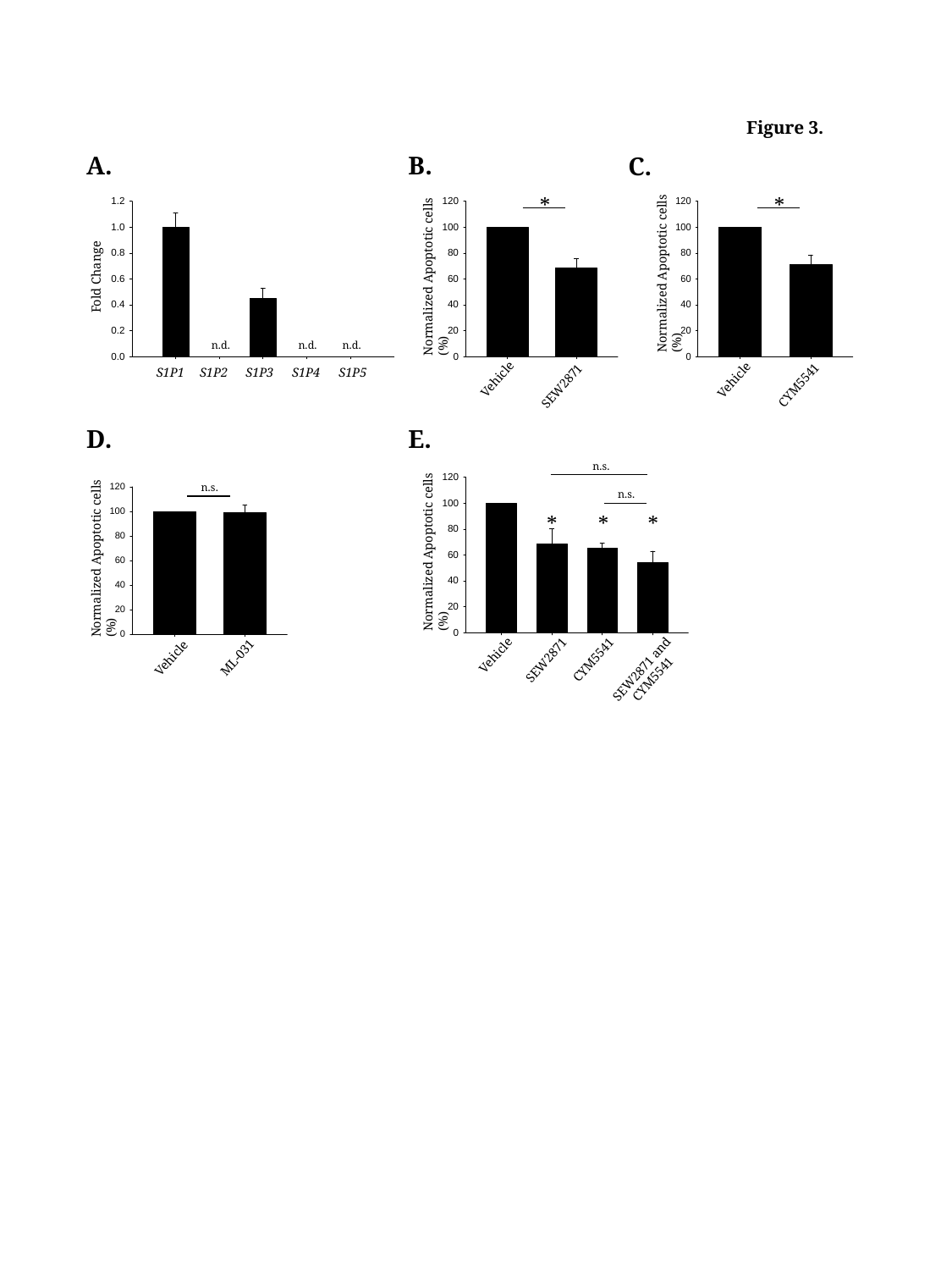

Figure 3.
A.
B.
C.
*
*
Normalized Apoptotic cells (%)
Normalized Apoptotic cells (%)
Fold Change
n.d.
n.d.
n.d.
Vehicle
Vehicle
CYM5541
S1P4
S1P3
S1P2
S1P1
S1P5
SEW2871
D.
E.
n.s.
n.s.
n.s.
*
*
*
Normalized Apoptotic cells (%)
Normalized Apoptotic cells (%)
Vehicle
ML-031
Vehicle
CYM5541
SEW2871
SEW2871 and CYM5541

## Slide 4
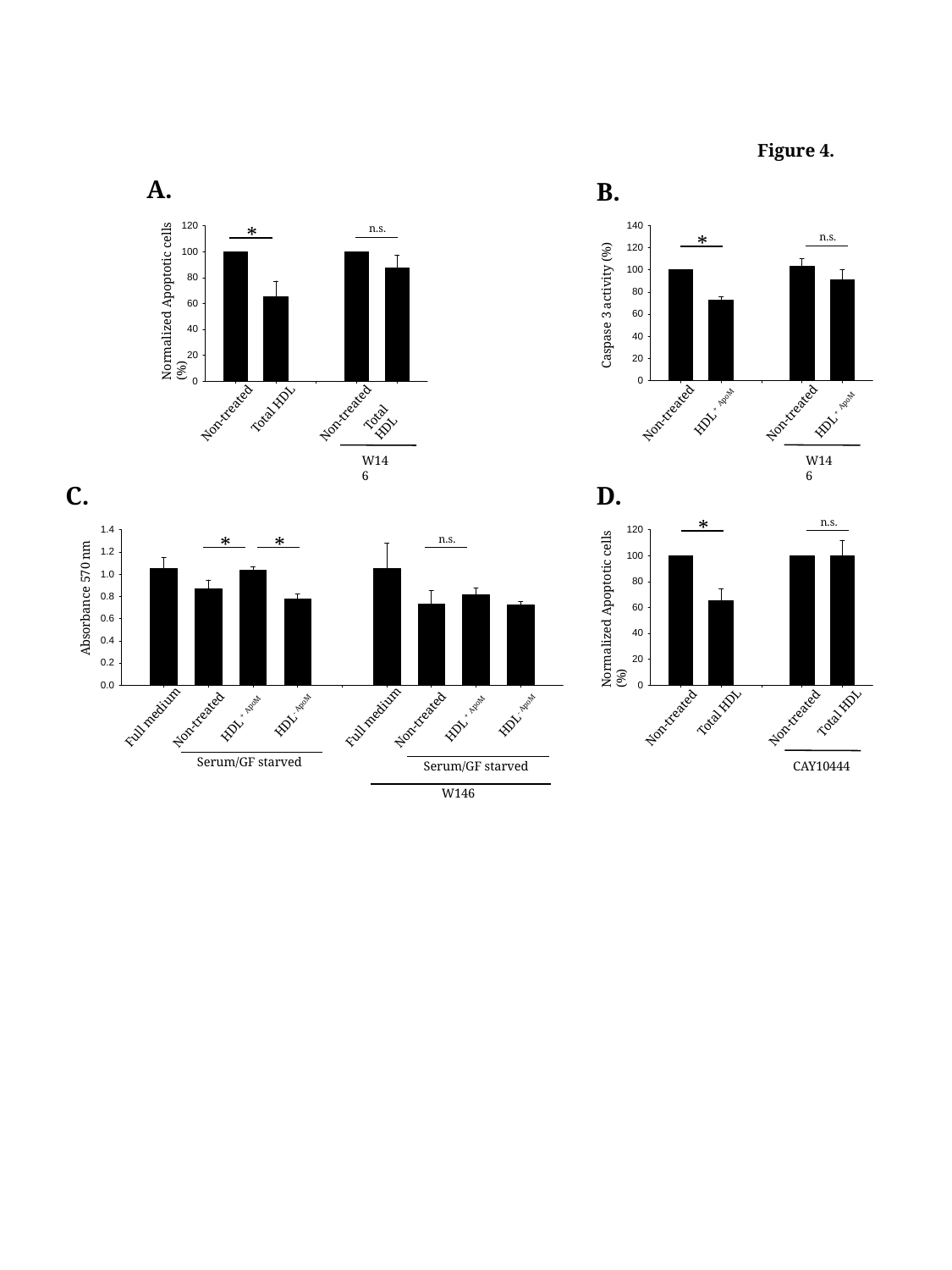

Figure 4.
A.
B.
n.s.
*
n.s.
*
Normalized Apoptotic cells (%)
Caspase 3 activity (%)
HDL + ApoM
HDL + ApoM
Total HDL
Total HDL
Non-treated
Non-treated
Non-treated
Non-treated
W146
W146
C.
D.
n.s.
*
*
*
n.s.
Absorbance 570 nm
Normalized Apoptotic cells (%)
Total HDL
Total HDL
HDL + ApoM
HDL + ApoM
HDL- ApoM
HDL- ApoM
Non-treated
Non-treated
Non-treated
Non-treated
Full medium
Full medium
Serum/GF starved
CAY10444
Serum/GF starved
W146

## Slide 5
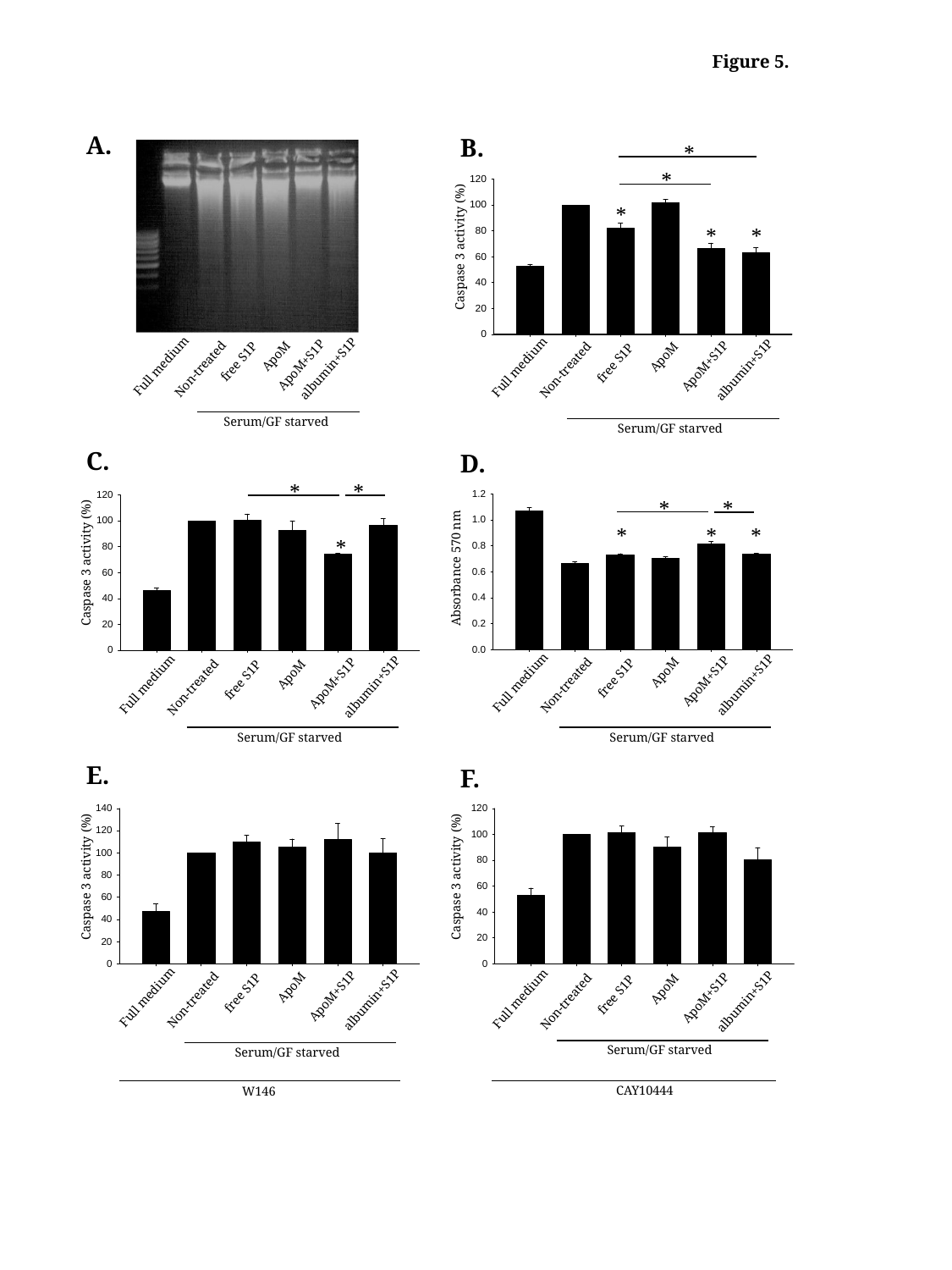

Figure 5.
A.
B.
*
*
*
*
*
Caspase 3 activity (%)
ApoM
free S1P
ApoM+S1P
albumin+S1P
Non-treated
Full medium
ApoM
free S1P
ApoM+S1P
albumin+S1P
Non-treated
Full medium
Serum/GF starved
Serum/GF starved
C.
D.
*
*
*
*
*
*
*
*
Caspase 3 activity (%)
Absorbance 570 nm
ApoM
free S1P
ApoM+S1P
albumin+S1P
Non-treated
Full medium
ApoM
free S1P
ApoM+S1P
albumin+S1P
Non-treated
Full medium
Serum/GF starved
Serum/GF starved
E.
F.
Caspase 3 activity (%)
Caspase 3 activity (%)
ApoM
free S1P
ApoM+S1P
albumin+S1P
Non-treated
Full medium
ApoM
free S1P
ApoM+S1P
albumin+S1P
Non-treated
Full medium
Serum/GF starved
Serum/GF starved
CAY10444
W146

## Slide 6
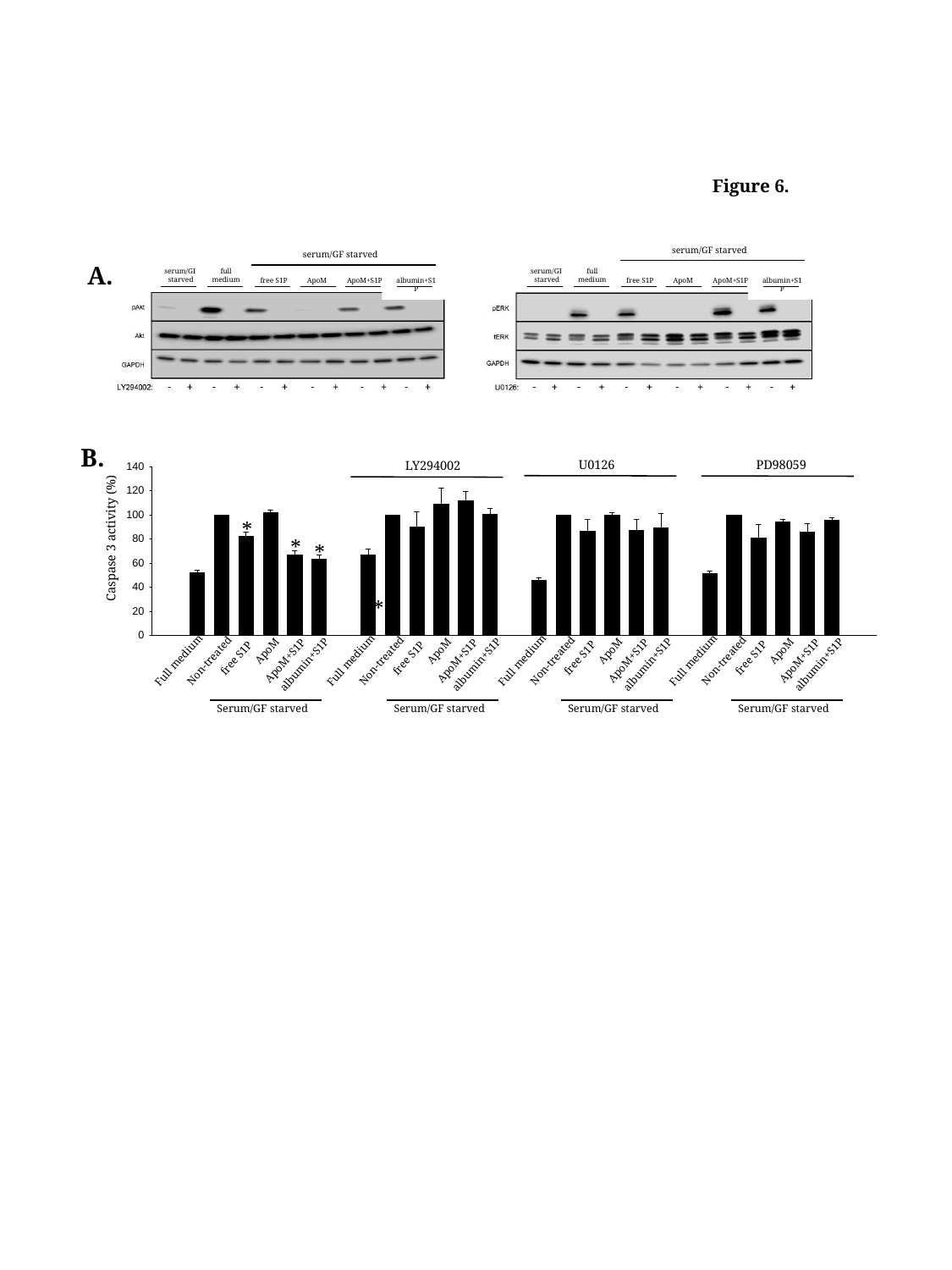

Figure 6.
serum/GF starved
serum/GF
starved
full medium
free S1P
ApoM
ApoM+S1P
albumin+S1P
serum/GF
starved
full medium
free S1P
ApoM
ApoM+S1P
albumin+S1P
serum/GF starved
A.
serum/GF
starved
full medium
free S1P
ApoM
ApoM+S1P
albumin+S1P
B.
U0126
PD98059
LY294002
*
Caspase 3 activity (%)
*
*
ApoM
free S1P
ApoM+S1P
albumin+S1P
Non-treated
Full medium
ApoM
free S1P
ApoM+S1P
albumin+S1P
Non-treated
Full medium
ApoM
free S1P
ApoM+S1P
albumin+S1P
Non-treated
Full medium
ApoM
free S1P
ApoM+S1P
albumin+S1P
Non-treated
Full medium
*
Serum/GF starved
Serum/GF starved
Serum/GF starved
Serum/GF starved

## Slide 7
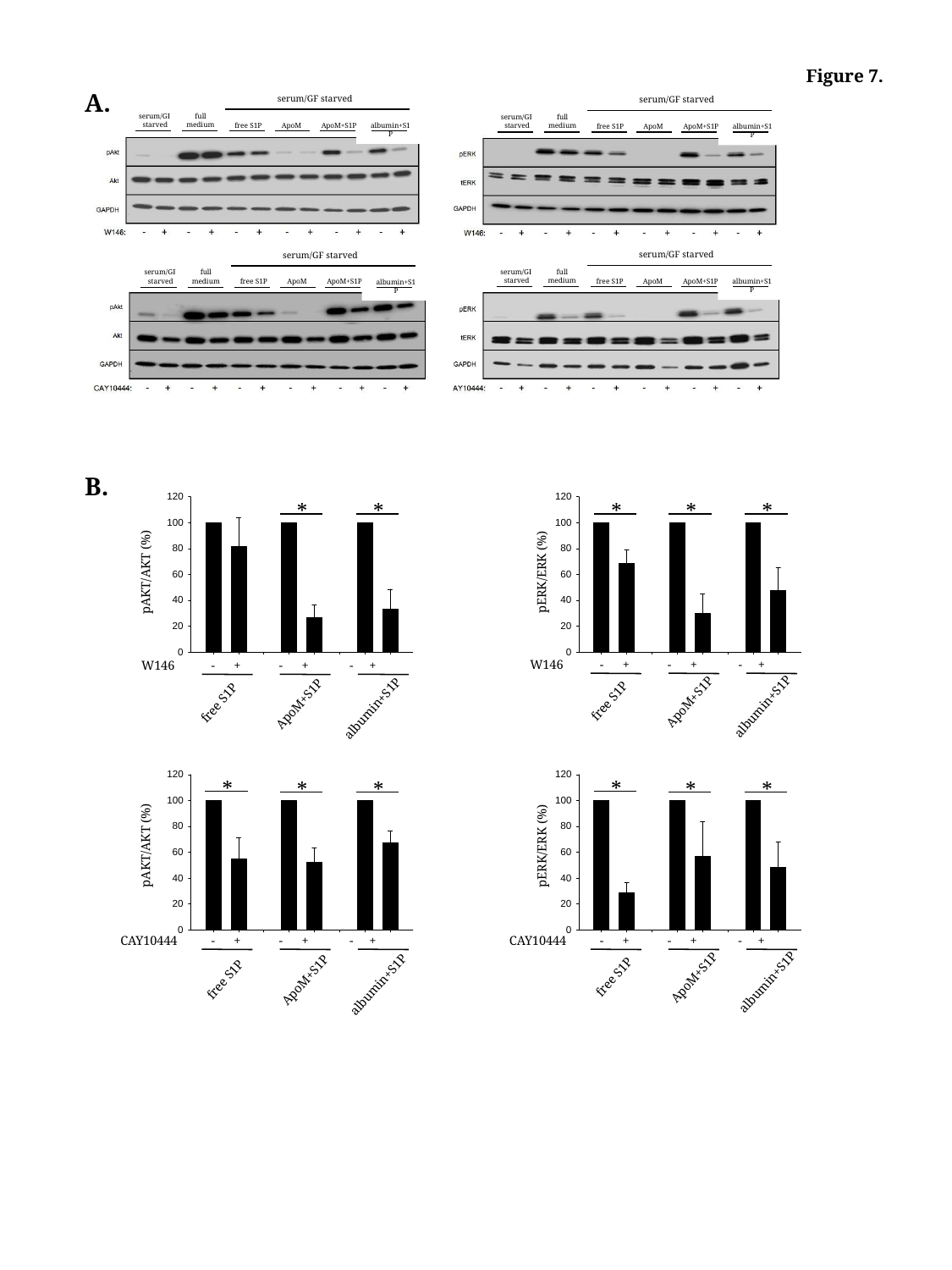

Figure 7.
A.
serum/GF starved
serum/GF
starved
full medium
free S1P
ApoM
ApoM+S1P
albumin+S1P
serum/GF starved
serum/GF
starved
full medium
free S1P
ApoM
ApoM+S1P
albumin+S1P
serum/GF starved
serum/GF
starved
full medium
free S1P
ApoM
ApoM+S1P
albumin+S1P
serum/GF starved
serum/GF
starved
full medium
free S1P
ApoM
ApoM+S1P
albumin+S1P
B.
*
*
*
*
*
pAKT/AKT (%)
pERK/ERK (%)
- + - + - +
W146
- + - + - +
W146
free S1P
free S1P
ApoM+S1P
ApoM+S1P
albumin+S1P
albumin+S1P
*
*
*
*
*
*
pAKT/AKT (%)
pERK/ERK (%)
- + - + - +
- + - + - +
CAY10444
CAY10444
free S1P
free S1P
ApoM+S1P
ApoM+S1P
albumin+S1P
albumin+S1P

## Slide 8
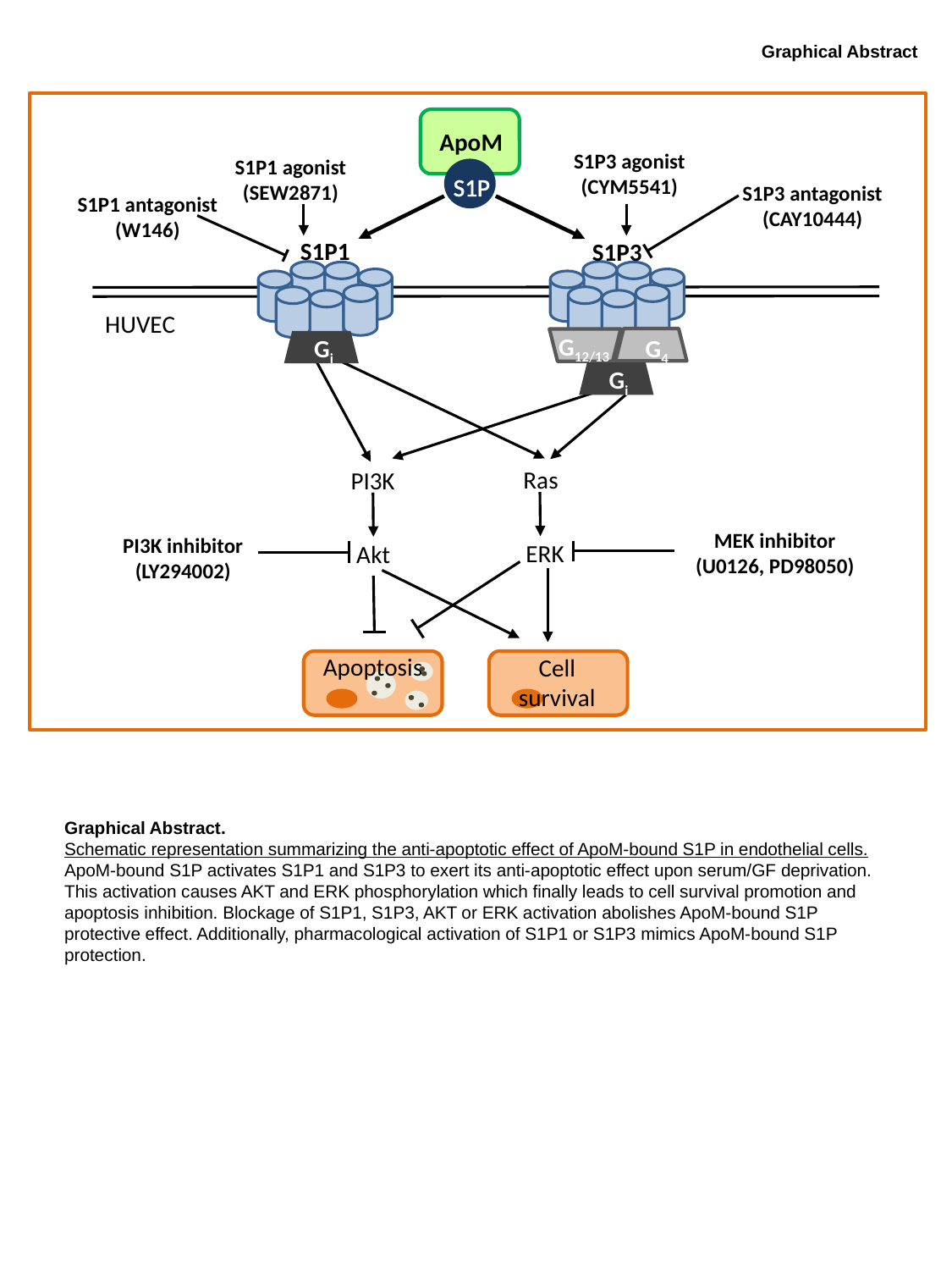

Graphical Abstract
ApoM
S1P
S1P3 agonist (CYM5541)
S1P1 agonist (SEW2871)
S1P3 antagonist (CAY10444)
S1P1 antagonist (W146)
S1P1
S1P3
HUVEC
G12/13
G4
Gi
Gi
Ras
PI3K
MEK inhibitor (U0126, PD98050)
PI3K inhibitor (LY294002)
ERK
Akt
Apoptosis
Cell survival
Graphical Abstract.
Schematic representation summarizing the anti-apoptotic effect of ApoM-bound S1P in endothelial cells.
ApoM-bound S1P activates S1P1 and S1P3 to exert its anti-apoptotic effect upon serum/GF deprivation. This activation causes AKT and ERK phosphorylation which finally leads to cell survival promotion and apoptosis inhibition. Blockage of S1P1, S1P3, AKT or ERK activation abolishes ApoM-bound S1P protective effect. Additionally, pharmacological activation of S1P1 or S1P3 mimics ApoM-bound S1P protection.
